# IMRT for head and neck cancer: reducing xerostomia and dysphagia

**DOI:** 10.1093/jrr/rrw047

**Published:** 2016-08-16

**Authors:** XiaoShen Wang, Avraham Eisbruch

**Affiliations:** 1 Department of Radiation Oncology, Cancer Hospital, Fudan University, 270 Dong'an Road, Shanghai, 200032, China; 2 Department of Radiation Oncology, University of Michigan, 1500 East Medical Center Drive, UH B2C490, Ann Arbor, Michigan 48109–0010, USA

## Abstract

Dysphagia and xerostomia are the main sequellae of chemoradiotherapy for head and neck cancer, and the main factors in reducing long-term patient quality of life. IMRT uses advanced technology to focus the high radiation doses on the targets and avoid irradiation of non-involved tissues. The decisions about sparing organs and tissues whose damage causes xerostomia and dysphagia depends on the evidence for dose–response relationships for the organs causing these sequellae. This paper discusses the evidence for the contribution of radiotherapy to xerostomia via damage of the major salivary glands (parotid and submandibular) and minor salivary glands within the oral cavity, and the contribution of radiotherapy-related effect on important swallowing structures causing dysphagia. Recommendations for dose limits to these organs, based on measurements of xerostomia and dysphagia following radiotherapy, are provided here.

## INTRODUCTION

Due to the complicated anatomic relationship between the tumor and normal structures in the head and neck (HN), and the importance of organ preservation in maintaining the patient's quality of life (QoL), considerations of intensifying therapy must be balanced with increased toxicity of intense treatment regimens. Radiotherapy (RT) has always played an important role in the treatment of head and neck cancers (HNCs) [1], and in recent years an increasing role for systemic chemotherapy and molecular targeted therapy for locally advanced disease has evolved [2–10]. Intensification of RT for locally advanced HNC has led to significantly improved locoregional control and survival compared with conventional RT [3–10]. However, these improvements are accompanied with increased toxicity [8–10].

Currently, besides improving tumor control rate, another important goal is to reduce the probability of radiation-induced complications in order to improve the survivors’ QoL. The application of 3D conformal radiation therapy (3D-CRT) and intensity-modulated radiotherapy (IMRT) signified a major improvement over conventional 2D RT. Using 3D treatment-planning systems (TPSs), both the target volume and organs at risk (OARs) can be contoured on the planning CT, and the spatial relationship between target volume and OARs can be clearly demonstrated in 3D. Using IMRT, the radiation beams can be optimized to deliver a higher dose to specified target volumes, while reducing the dose to adjacent OARs. Using IMRT to treat HNC is especially attractive due to its unique ability to treat the concave target shapes, the close vicinity of the targets and many dose-limiting and non-involved OARs, and because of the lack of breathing-related motion in these tumors.

 As IMRT allows highly conformal dose distributions to target volumes of almost any shape, appropriate selection and accurate delineation of the target volumes and the avoidable organs becomes of critical importance [11]. Over the past few years, some authors have made recommendation guidelines for selection of the clinical target volume (CTV) for both the primary tumors and neck nodal areas [12–16]. Besides appropriate selection of normal organs, the other important item is to set dose constraints when designing IMRT plans so as to spare OARs. At present, most available data about the tolerance dose of normal tissues are based on retrospective analyses or expert opinion [17, 18]. Because of these drawbacks, these analyses might not allow definitive conclusions.

Xerostomia and dysphagia are both the main acute and late complications that result in decreased QoL during and after radiotherapy. This chapter describes the efforts to prevent the above-mentioned therapy-related complications by presenting state of the art evidence regarding organ-sparing by advanced RT technology.

## XEROSTOMIA

Xerostomia (dry mouth) is the most common and prominent complication during and after radiotherapy for HNC as a result of damage to the salivary glands. Radiation-induced injury to the salivary glands alters the volume, consistency and pH of secreted saliva [19]. Because the severity of the damage to the salivary glands is dependent both on the total radiation dose and on the volume of irradiated tissue, current studies on organ-preserving RT have focused on sparing the salivary glands from unnecessary irradiation [20].

## PAROTID GLANDS

Limiting the volume of the parotid glands receiving a high radiation dose has long been recognized as a major factor in reducing the severity of xerostomia. For most HNCs, especially squamous cell carcinoma, the necessity of treating the bilateral level II lymph nodes makes it difficult to spare the parotid glands using standard, laterally opposed RT techniques. However, with 3D-CRT or IMRT, it is possible to partly spare at least one parotid gland in selected patients. A high dose is delivered to only a small part of the parotid gland that is located closest to the target volumes, while the rest of the parotid receives a low dose or no dose at all [20, 21]. Thus the salivary function is partially preserved and can increase over time via a compensatory response on the part of the parotid that received a low dose [20, 22].

Over the past 10 years, an increasing body of data has demonstrated the ability of 3D-CRT and IMRT to deliver dose distributions that allow partial preservation of parotid function, assessed by either salivary flow measurements or salivary gland scintigraphy (Table 1). A growing number of prospective clinical trials have demonstrated that parotid-sparing IMRT is sufficient to reduce long-term xerostomia without jeopardizing local-regional control for nasopharyngeal cancer (NPC) (Table 2). Although IMRT for HNC is promising in terms of local tumor control and improvement of salivary function according to single institution studies, these data need to be validated in randomized multi-institutional studies. Recently, several randomized clinical trials have further confirmed that IMRT for NPC could reduce the severity of xerostomia without jeopardizing the tumor control rate compared with conventional RT [39, 43, 44]. In oropharyngeal cancer, it has also been demonstrated that IMRT preserved salivary flow in two prospective multi-institutional studies [32, 33, 45].

**Table 1.** Overview of prospective trials on parotid-sparing radiotherapy

Author (year)	No.	Site	Stage	RT technique	Constraint (mean dose, Gy)	Objective endpoint	Subjective endpoint
Eisbruch (1996) [21]	15	All	I–IV	3D	21 ± 8	SF	XQ
Eisbruch (1999) [33]	88	All	I–IV	3D	≤26 (stimulated) ≤24 (unstimulated)	SF	NS
Chao (2001) [23]	41	All	II–IV	3D/IMRT	≤32	SF	XQ
Eisbruch (2001) [24]	84	All	I–IV	3D/IMRT	≤26	SF	XQ
Henson (2001) [25]	20	All	II–IV	3D	≤26	SF	NS
Maes (2002) [26]	39	All	I–IV	3D	≤20	SGS	VAS
Munter (2004) [27]	18	All	I–IV	IMRT	≤26	SGS	NS
Parliament (2004) [28]	23	All	I–IV	IMRT	≤26	SF	XQ
Saarilahti (2005) [29]	17	OP/NP	II–IV	IMRT	≤25.5	SF	NS
Blanco (2005) [30]	65	All	I–IV	3D/IMRT	≤25.8	SF	NS
Scrimger (2007) [31]	47	All	I–IV	IMRT	≤26	SF	XQ
Eisbruch (2010) [32]	69	OP	I-II	IMRT	<26	SF	XQ

No. = number, IMRT = intensity-modulated radiotherapy, SF = salivary flow, XQ = xerostomia questionnaire, NS = not stated, SGS = salivary gland scintigraphy, VAS = visual analogue scale, OP = oropharnyx, NP = nasopharynx, All = all subsites.

**Table 2.** Results of non-randomized studies on IMRT in the treatment of NPC

Author (year)	No.	Stages III + IV (%)	CT (%)	FU (months)	LRC/RC	OS	DMFS	Xerostomia (%)
Sultanem (2000) [34]	35	72	91	21.8	100 (4 y)	94 (4 y)	57 (4 y)	(At 2 years) Grade 0: 50, Grade 1: 50
Lee (2002) [35]	67	70	75	31	98 (4 y)	88 (4 y)	66 (4 y)	(At 2 years) Grade 0: 66, Grade 1: 32, Grade 2: 2
Kam (2004) [36]	63	57	30	29	92 (3 y)	90 (3 y)	79 (3 y)	(At 2 years) Grade 1–2: 23
Wu (2006) [37]	75	56	NA	23.8	87 (2 y)	87 (2 y)	82 (2 y)	(At 39 months) Grade 1: 24, Grade 2: 18.6, Grade 3: 1
Wolden (2006) [38]	74	77	93	35	91 (3 y)	83 (3 y)	78 (3 y)	(At 1 year) Grade 0: 25, Grade 1: 42, Grade 2: 32
Lee (2009) [39]	68	59	84	31	93 (2 y)	80 (2 y)	85 (2 y)	(At 1 year) Grade 2: 13.5, Grade 3: 3.1
Tham (2009) [40]	195	63	57	36.5	93 (3 y)	94.3 (3 y)	89.2 (3 y)	Grade 0–2:97, Grade 3: 3
Lin (2009) [41]	323	80.5	91.3	30	95 (3 y)	90 (3 y)	90 (3 y)	(At 24 months) Grade 0: 5.4, Grade 1: 86.8, Grade 2: 7.8
Lin (2009) [42]	370	83.2	90.3	31	95 (3 y)	86 (3 y)	89 (3 y)	(At 24 months) Detectable xerostomia: 7.8%, Grade 3–4: 0

No. = number of patients, CT = chemotherapy, FU = follow-up, LRC/RC = locoregional control/regional control, OS = overall survival, DMFS = distant metastatic-free survival, NA = not available, y = year.

The practice guidelines must be made for appropriate preservation of the parotid function, because overemphasis on parotid sparing might lead to geographical miss and unexpected patterns of failure. In recent years, emerging data on locoregional failures after 3D-CRT or IMRT has facilitated the development of practice guidelines for parotid-sparing IMRT for HNC. In patients with negative lymph nodes, at least one, but usually both, parotid glands can be safely spared, depending on the location of the primary cancer. In patients with unilateral neck disease, sparing of the contralateral parotid gland dose not result in increased marginal failures [46, 47]. However, sparing of the ipsilateral parotid gland should be given lower priority, especially if there are involved lymph nodes at level II [14, 20, 48]. In patients with extensively involved bilateral nodal disease, meaningful preservation of the parotid function should never be considered at the cost of underdosing the target volume, because locoregional failure is the worst treatment outcome. In addition, more detailed proposals have been given about the cranial border of level II, as it has clear relevance to the possibility of sparing the parotid [49]. For patients without nodal disease, the upper boundary of level II is placed at the caudal edge of the lateral process of the first vertebra [16]. For patients with involved nodal disease, level II on the involved neck side is extended to the skull base and includes the retrostyloid space [50].

 Nowadays, definition of dose/volume–response relationships for the parotid glands has been well established from the data regarding correlation of residual salivary function with radiation dose. The consensus has been reached that xerostomia can be substantially reduced by limiting the mean parotid gland dose to <26–30 Gy as a planning criterion [51]. By reducing the mean dose to at least one parotid gland, salivary function can be partially preserved, and it improves gradually over time. Thus both the prevalence and extent of dry mouth can be greatly reduced over time. This effect has been demonstrated in several clinical studies [25, 28, 37, 43]. However, the improvement in objective parotid function as measured by salivary flow is not always accompanied with improved patient-reported xerostomia [28, 31, 44]. One study indicated that the observer-based grades underestimated the severity of xerostomia compared with the patient self-reported scores [52]. We suggest that not only the objective parotid function, but also patient's subjective scores should be the main end points in evaluating xerostomia. Because xerostomia is mainly an issue of QoL, symptoms reported by patients are more suggestive of its true severity.

## SUBMANDIBULAR GLANDS

Under stimulated status, 60–65% of saliva is produced by the parotid glands, 20–30% by the submandibular glands (SMGs), and 2–5% by the sublingual glands. However, in the non-stimulated state, the SMGs contribute up to 90% of the salivary output [53]. Moreover, the saliva secreted by the parotid glands is purely serous, whereas saliva from the SMGs also contains mucins, which chiefly contribute to the patient's subjective sense of moisture [20]. Therefore, it is also important to protect the function of the SMGs.

One study demonstrated that by surgical transfer of the SMGs to the submental space before RT, thus avoiding them being irradiated, can significantly prevent xerostomia, confirming the important role of the SMGs [54]. However, this surgical technique has not been widely applied due to its drawbacks. It is reasonable to infer that the severity of xerostomia can be reduced by sparing the SMGs from radiation. A prospective non-randomized study has revealed the feasibility of sparing the SMGs without compromising local tumor control [55].

The data regarding dose–response relationships of the SMGs came from Tsujii [56]. He used 99mTc-pertechnetate scintigraphy to measure salivary gland function and reported an unexpected improvement in SMG function as the dose increased from 10 to 30 Gy, followed by a steep decline after 50 Gy. He also demonstrated that the parotid glands were more sensitive to radiation than the SMGs at 0–3 months following 20–70 Gy. Recently, the dose–response relationship for the SMGs has been established on the basis of patients who underwent salivary flow measurements selectively from Wharton's duct before and after RT. The function of the SMGs was shown to be dependent on the mean radiation dose, with recovery over time up to a mean dose of 39 Gy [57]. A recent study showed clinical benefit from sparing the contralateral SMGs [58].

Reduction of the radiation dose to the SMG might be potentially dangerous owing to its close proximity to the base of tongue, tonsil, and level IIa lymph nodes. Therefore, when trying to preserve the function of the SMGs, we must take into account the potential risk of reducing local regional tumor control. At present, available evidence regarding the efficacy and safety of SMGs-sparing IMRT is extremely limited.

## ORAL CAVITY AND MINOR SALIVARY GLANDS

The minor salivary glands, which are dispersed throughout the oral cavity, produce up to 70% of the total mucins secreted by the salivary glands [55]. Thus it is reasonable to anticipate that limiting the radiation dose to the oral cavity might contribute to the reduction of patient-reported xerostomia. Moreover, sparing the oral cavity from unnecessary radiation might have additional benefits in preventing mucositis and taste loss [59]. Therefore, the uninvolved oral cavity should be delineated as an OAR, and be given dose constraint in designing the IMRT plan whenever possible. At present, the mean non-involved oral cavity dose was set to be ≤30 Gy in the Department of Radiation Oncology, University of Michigan, although with very low priority.

## DYSPHAGIA

Dysphagia has a devastating effect on patient daily life, and can even lead to life-threatening complications, such as aspiration pneumonia [60]. Radiotherapy for HNC inevitably results in appreciable dose delivery to some of the critical structures necessary for normal deglutition, such as the tongue, soft palate, and pharyngeal and laryngeal muscles, which leads to unavoidable mucositis and swallowing difficulty [8–10].

Dysphagia can be evaluated by both objective and subjective methods. As for xerostomia, one study also indicated that patient-reported symptoms were not representative of findings from objective evaluation of swallowing [61]. To date, many researchers have carried out clinical trials to analyze the relationship between irradiated structures and dysphagia, and the findings of published studies are nearly consistent regarding the crucial structures associated with swallowing dysfunctions (Table 3). Both the mean dose to the pharyngeal constrictor muscles and the larynx, as well as the volume of these structures receiving 50–60 Gy, have been shown to correlate remarkably with the prevalence of dysphagia [61–70]. These findings imply that limiting the dose to the crucial swallowing structures might decrease both the incidence and severity of radiation-induced dysphagia.

**Table 3.** Overview of studies assessing crucial structures for late dysphagia

Author (year)	Sample	Site	Dysphagia endpoint	Dosimetric factors correlated with dysphagia
Feng (2007) [61]	36	OP/NP	VF, UW QOL	PCMs (mean dose, V50, V60, V65) and larynx (mean dose, V50)
Levendag (2007) [62]	56	OP	H&N 35	Superior and middle PCMs (mean dose)
Jensen (2007) [60]	25	Pharynx	H&N 35	Supraglottic larynx (mean dose, median dose, V60, V65)
Teguh (2008) [63]	81	OP/NP	H&N 35	Superior and middle PCMs (mean dose)
Teguh (2008) [64]	20	OP	FEES	Superior PCMs (mean dose)
Caglar (2008) [65]	96	All	VF	Inferior PCMs (mean dose, V50, D60) and larynx (mean dose, V50, D60)
Caudell (2009) [66]	83	All	VF	Inferior PCMs (V60, V65) and larynx (mean dose, V55, V60, V65, V70)
Dirix (2009) [67]	53	All	H&N 35	Middle PCMs (mean dose, V50) and supraglottic larynx (mean dose)
Feng (2010) [68]	73	OP	VF, UW QOL	PCMs (mean dose, V50, V60, V65) and larynx (mean dose, V50)
Eisbruch (2004) [69]	26	All	VF	PCMs (V50) and the glottic and supraglottic larynx (V50)

OP = oropharnyx, NP = nasopharynx, VF = videofluoroscopy, UW QOL = University of Washington Quality of Life Scale, PCMs = pharyngeal constrictor muscles, V50 = volume receiving ≥50 Gy, V60 = volume receiving ≥60 Gy, V65 = volume receiving ≥65 Gy, H&N 35 = EORTC Head and Neck 35 swallowing symptom score, FEES = fiberoptic endoscopic evaluation of swallowing, All = all subsites, D60 = minimum dose received by 60% of a structure, V70 = volume receiving ≥70 Gy.

In order to reduce dysphagia using IMRT, it is important to identify and delineate the dysphagia- and aspiration-related structures (DARS). Eisbruch *et al*. [70] first reported that radiation damage to the pharyngeal constrictors and the glottic/supraglottic larynx were implicated in post-RT dysphagia. They suggested that reducing the radiation dose to the DARS may lead to improved swallowing outcomes. Following this, a series of trials have been initiated to establish whether dose reduction to DARS can improve swallowing outcomes for HNC treated by IMRT. The results of these studies are consistent and show that increased radiation dose to a larger volume of the pharyngeal constrictors results in worse dysphagia [61–71]. A dose–risk ratio has been suggested by several investigators. Levendag *et al*. [63] reported a 19% increase in the probability of dysphagia with every additional 10 Gy to the superior and middle constrictor muscles. Li *et al*. [71] suggested that in order to reduce the risk of prolonged gastrostomy feeding tube use, the dose constraint should be a mean dose of <55 Gy to the inferior constrictor muscle, and a maximum dose of <60 Gy to the cricopharyngeal inlet.

However, no clear volume or dose constraints can be determined from the current literature. At present, the best way is to keep the radiation dose to these structures as low as possible. Prospective, longitudinal studies, including baseline evaluation with pre-determined follow-up assessment at different time points, are still needed to better understand the relationship between dose/volume and swallowing outcomes. In Feng's study, significant correlations were observed between aspirations and the mean doses to the pharyngeal constrictor (PC) and glottic supraglottic larynx (GSL), as well as the partial volumes of these structures receiving 50–65 Gy [62]. Using these dose–volume parameters as initial IMRT optimization goals, a prospective clinical trial was carried out, and the results suggest that chemo-IMRT aiming to reduce dysphagia can be performed safely for oropharyngeal cancer [69]. At present, we try to keep the mean dose to the non-involved PC and GSL ≤50 Gy at the University of Michigan. However, avoiding underdosing to the targets in the vicinity of the swallowing structures should be the highest priority.

Another approach to sparing the swallowing structures is selective delineation of the nodal volume, especially avoiding the delineation of the medial retropharyngeal lymph nodes. These nodes are located between the PC muscles and the prevertebral fascia near the midline, and their exclusion from the elective nodal target volume might significantly contribute to sparing the PC muscles [20, 62].

Recently, a multicenter prospective study demonstrated that RT in conjunction with cetuximab improved tumor control without increasing common RT-associated toxicities, such as dysphagia, and did not have a negative effect on patients’ QoL compared with RT alone [4, 72]. Therefore, it is inferred that cetuximab could potentially decrease treatment-related toxicity by replacing more toxic chemotherapy without jeopardizing survival. However, to date, there are no phase III clinical trials that directly compare cetuximab and RT to standard chemotherapy and RT. Moreover, cetuximab is not without toxicity. According to a retrospective study, concomitant cetuximab with IMRT resulted in an ∼10-fold increase in the rate of Grade 3/4 transient dermatitis compared with the use of concomitant cisplatin (34% vs 3%) [73]. The currently available data are insufficient to warrant changing the standard practice of concurrent chemoradiotherapy to cetuximab and RT in order to reduce dysphagia.

## CONCLUSIONS

QoL may be improved by the application of IMRT without compromising tumor control for HNC. When treating HNC with IMRT or 3D-CRT, it is important to contour the target volume accurately, as well as to delineate all relevant normal structures at risk, and the available radiation-dose constraints must be taken into account. Currently, xerostomia can be successfully prevented or reduced by restricting the maximum mean dose threshold to 26 Gy for at least one parotid gland as well as making an effort to reduce the doses to the contralateral SMG and to the minor salivary glands in the oral cavity. Late dysphagia can be reduced by keeping the mean dose to the non-involved PC muscles and the larynx ≤50 Gy. However, prospective collection of dosimetric data, along with the corresponding functional outcomes, is still needed in order to establish more precise dose–response curves.
